# A Water-Based Sequential Preparatory Approach vs. Conventional Aquatic Training in Stroke Patients: A Randomized Controlled Trial With a 1-Month Follow-Up

**DOI:** 10.3389/fneur.2020.00466

**Published:** 2020-06-18

**Authors:** Giulia Temperoni, Andrea Curcio, Marco Iosa, Marco Antonio Mangiarotti, Daniela Morelli, Sara De Angelis, Serena Vergano, Marco Tramontano

**Affiliations:** ^1^Fondazione Santa Lucia IRCCS, Rome, Italy; ^2^ANIK, Associazione Nazionale Idrokinesiterapisti, Rome, Italy

**Keywords:** aquatic therapy, water-based therapy, stroke, balance, gait, spasticity

## Abstract

**Background:** Many studies hypothesize that people who have suffered stroke could benefit from water-based exercises to improve their strength and ability to perform the activities of daily living.

**Objective:** The study aim was to compare the effects of a water-based sequential preparatory approach (SPA) and conventional aquatic therapy in improving motor functions and quality of life in patients with chronic stroke.

**Methods:** Thirty-three chronic stroke outpatients diagnosed with hemiplegia were recruited and randomly assigned to the experimental or control group. Subjects in the experimental group underwent a trial water-based SPA balance training, and patients in the control group were given traditional water balance training. Both groups of participants underwent 45 min of therapy twice a week for 4 weeks. All patients were evaluated before treatment (T0), after 4 weeks of training (T1), and 4 weeks after the end of training (T2) using the Berg balance scale (BBS), the modified Barthel index (MBI), the Tinetti balance and gait scale (TBG), the Stroke Specific Quality Of Life Scale (SS-QOL), and the modified Ashworth scale (MAS).

**Results:** After the training, statistically significant differences (*p* < 0.05) were found between the groups in their score averages on the BBS (*p* = 0.01) and the SS-QOL scale (*p* = 0.03). Furthermore, the SPAg showed a significantly greater percentage of improvement on the BBS (*p* = 0.02) and the SS-QOL (*p* = 0.03). Both groups obtained a significantly improved MAS score (*p* < 0.01).

**Conclusion:** Results indicate that water training based on an SPA is more effective than traditional aquatic training for balance rehabilitation of chronic poststroke patients.

## Introduction

Many stroke patients have sensorimotor impairments that disrupt their motor performance (1). Furthermore, stroke-induced brain damage often results in balance and gait disorders that can significantly affect quality of life (QoL) (2). The 2016 SPREAD guidelines (3) documented severe disability in 40% of stroke patients that persisted even if they had received a specific rehabilitation training within the first 6 months following the event. Although many studies have documented gait recovery in hemiparetic patients within 6 months after a brain stroke (4, 5), residual balance and gait disorders are common in the poststroke chronic phase (6). Balance and gait are complex multifactorial systems in which motor, sensory, and cognitive components interact. This information is integrated by the central nervous system into a continuous sensorial re-weighting that ensures postural control in both static and dynamic conditions (7, 8). The weighting of the sensory inputs likely depends on environmental conditions, and it changes according to the motor task being performed by the subject (9–11). The integration of multisensory information (i.e., visual, vestibular, and somatosensory) is impaired following a stroke (12).

A Cochrane Review (13) systematically synthesized and compared the effects of aquatic and land-based therapies on the activities of daily living (ADLs) of patients following stroke and found that water-based exercises improved strength and ADLs (13). A recent review (14) indicates that randomized controlled trials (RCTs) comparing aquatic methods in both environments are lacking. The same movements in water and on dry land that target postural stability and gait require different competences. For example, the postural instability that occurs while squatting in water was enacted on land by sitting on a therapy ball (15). Furthermore, land-based conventional rehabilitation is generally task oriented, customized, and challenging and follows a specific preparatory sequence of exercises according to patients' disabilities (16). The water-based exercise protocol reported in previous reviews consisted of walking, stretching, balance, and aerobic exercises that were not systematically organized (17–24). Overall, the research methodology was lacking in the follow-up, and the long-term effects are still not clear (14). Therefore, we believe that a sequential preparatory approach (SPA), based on increasing difficulty and following a specific sequence of preparatory exercises (from the simplest to the most complex), should also be used in an aquatic environment.

Based on the above line of reasoning, our hypothesis is that a water-based SPA might be more effective than conventional aquatic therapy. For these reasons, the aim of this study is to investigate the effects of a water-based SPA compared with a conventional water-based therapy on motor functions and QoL in stroke survivors.

## Methods

### Trial Design

**Figure 1:**
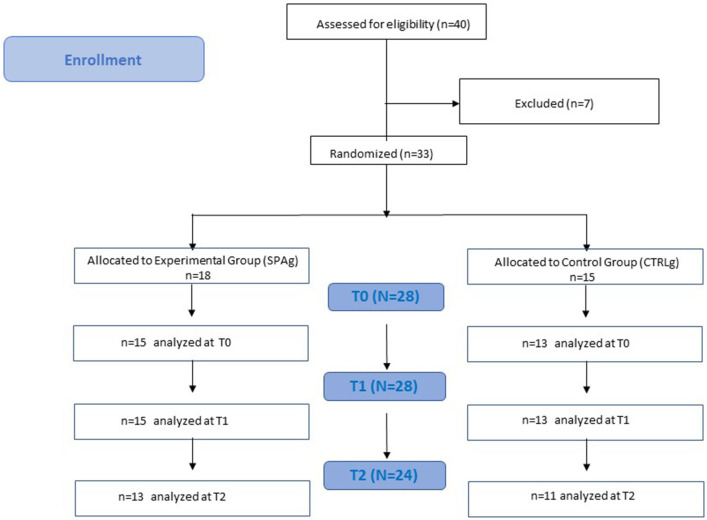
Flowchart study design. Clinical evaluation was performed at baseline (T0), after 4 weeks of treatment (T1), and 4 weeks after the end of treatment (T2). Both groups performed the training twice a week for 4 weeks in a rehabilitation pool of the Fondazione Santa Lucia (FSL) Hospital.

This was a two-arm, single-blind randomized controlled trial with a 1-month follow-up (Figure 1). The aim was to investigate the effects of a water-based, sequential, preparatory approach compared with a conventional, water-based therapy in stroke survivors. The guidelines for Good Clinical Practice, and the Consolidated Standards of Reporting Trials (CONSORT), were followed. This trial was approved by the Local Ethics Committee of Fondazione Santa Lucia (Protocol CE/PROG.728); all participants gave their written informed consent for participation in the study.

A researcher who was not involved in the intervention sessions assessed the patients' eligibility to participate on the basis of the inclusion and exclusion criteria. Participants were randomly assigned to one of two groups: experimental (SPAg) or control (CTRLg) group.

### Participants

Thirty-three outpatients (21 males, mean age 51 years) with a diagnosis of stroke (>6 months after stroke) were recruited and enrolled on the basis of consecutive sampling through the Aquatic Rehabilitation Services of Fondazione Santa Lucia (FSL), Institute for Research and Health Care, from February 2019 to September 2019. Inclusion criteria were stroke with unilateral hemiplegia within the previous 6 months and ability to walk without any device or need of continuous physical assistance to support body weight or maintain balance (Functional Ambulation Classification ≥ 3) and acclimatization to water. Subjects were aged between 25 and 80 years. Exclusion criteria were cognitive deficits affecting the ability to understand task instructions (Mini-Mental State Examination > 24), severe unilateral spatial neglect (diagnosed with a test battery that included the Letter Cancellation test, Barrage test, Sentence Reading test, and the Wundt-Jastrow Area Illusion Test), severe aphasia (diagnosed by means of neuropsychological assessment), presence of other neurological diseases, presence of cutaneous and mycosis infections, presence of open wounds, eczema, skin ulcers, and decubitus lesions, presence of severe burns, presence of percutaneous endoscopic gastrostomy (PEG) or tracheostomy, urinary incontinence, and presence of otitis and orthopedic or cardiac comorbidities that would limit participation in the experimental and conventional training (all of which were clinically evaluated).

Demographic characteristics of the sample are reported in Table 1.

**Table 1:** Demographic characteristics.

	**SPAg**	**CTRLg**
Age (years)	52.44 ± 10.51	52.01 ± 17.10
Gender	14M	7M
Time since stroke (months)	13.33 ± 7.10	16.87 ± 13.79
Stroke location (emisphere)	8R	8R
Stroke type, ischemic	7I	8I

*Mean ± standard deviation*.

*M, male; F, female; R, right; I, ischemic*.

### Interventions

Patients underwent eight individual rehabilitation sessions as outpatients (2 days/week, 4 weeks), in a rehabilitation pool at Fondazione Santa Lucia Neurorehabilitation hospital. Each session lasted 45 min. The water temperature was between 30 and 32°C.

### Sequential Preparatory Approach

The experimental training consisted of a sequential, preparatory approach aimed at enhancing dynamic postural stability. The exercises followed a specific sequence starting from a kneeling position, proceeding to a sitting position, and ending with a supine position. Step exercises preparatory for gait were performed using a step and two floating aids. Gait exercises were performed first with the upper limbs placed on two floating aids and then during a dual motor task (i.e., catching a ball thrown by the therapist). The training was carried out by two physiotherapists who had at least 5 years of experience in aquatic neurorehabilitation.

### Standard Aquatic Therapy

The control group performed water-based exercises, in line with suggestions of the Hydrotherapy Association of Chartered Physiotherapists Guidance on Good Practice in Hydrotherapy (25). These consisted of warm-up exercises, stretching exercises for the lower limbs, recruitment exercises, and walking exercises during each phase of gait (single stance, swing, and double stance). The training was carried out by the same physiotherapists who implemented the SPA.

### Outcomes

At enrollment, clinical and demographic data were collected. A blinded examiner assessed primary and secondary outcomes before treatment (T0), after 4 weeks of training (T1), and 4 weeks after the end of training (T2). The primary outcome measure was the Berg balance scale (BBS) (26).

Secondary outcome measures were the modified Barthel index (BIM) (27), the Tinetti balance and gait scale (TBG) (28), and the Stroke Specific Quality Of Life Scale (SS-QOL) (29). To assess potential side effects on spasticity, the modified Ashworth scale (MAS) (30) score was used to evaluate spasticity of elbow flexors, wrist flexors, and finger flexors of the affected upper limb and of hip adductors, knee extensors, and ankle plantar flexors of the affected lower limb. To facilitate data analysis, MAS scores (0, 1, 1+, 2, and 3) were assigned numerical values (0, 1, 2, 3, and 4, respectively). All clinical scale scores were collected by a physiotherapist who was blind to group allocation.

### Sample Size

The sample size complied with the minimum number of participants recommended by a power analysis performed on preliminary data (α = 0.05; β = 0.8; Effect size = 0.5) for nonparametric between-group comparisons (31). This sample-size estimation procedure recommends that at least 15 patients be included in each group (32).

### Blinding

A researcher who was not involved in the intervention sessions carried out the randomization.

Block randomization was performed with a computer-generated randomization list using a block size. Allocation concealment was ensured by using opaque envelopes. The researcher responsible for the randomization process deposited the list in a secure web-based storage.

### Statistical Analysis

An intention-to-treat protocol was adopted. Therefore, independently of the number and duration of the sessions actually performed, the patients remained in the same group they were originally assigned to and were not dropped. Complete case analysis was performed, and only the patients who were not re-assessed at T1 or T2 were considered dropouts.

IBM SPSS Statistics software (v23, IBM Corp., Armonk, NY, USA) was used. Data were reported in terms of means and standard deviations. For the descriptive analysis, we calculated the effectiveness of the increments of the scales used [(T1 score – T0 score/maximal score – T0 score) × 100] and [(T2 score – T0 score/maximum score – T0 score) × 100] (33). The Mann–Whitney *U*-test was used to compare data between groups at T0, T1, and T2. The Friedman test and the Wilcoxon signed-ranks test were used for within-subjects comparison for both groups at times T0–T1 and T0–T2.

## Results

Thirty-three patients met the inclusion criteria and were enrolled in the study. Five patients were released before the end of the training because of limited assistance, and four patients dropped out at T2 because they were involved in a new rehabilitation program (Figure 1). Statistical analysis was performed using the data of 28 patients who completed the evaluations at T1 (SPAg = 15; CTRLg = 13) and using the data of 24 patients who completed the evaluations at T2 (SPAg = 13; CTRLg = 11). As reported in Table 2, both groups showed significant improvement in their scores on all clinical scales used in the within-subjects comparison over time. Significant differences were found for the BBS score at T1 (*p* = 0.01), MAS scores for upper and lower limbs (*p* < 0.01), and the SS-QOL at T2 (*p* < 0.05) in the between-subjects analysis of the SPAg with respect to the CTRLg.

**Table 2:** Clinical scales scores.

	**SPAg**			**CTRLg**		
	**T0** **Mean ± SD**	**T1** **Mean ± SD**	**T2** **Mean ± SD**	**T0** **Mean ± SD**	**T1** **Mean ± SD**	**T2** **Mean ± SD**
MBI	83.9 ± 7.9	93.4 ± 10.3	98.2 ± 7.6	86 ± 13.4	90.9 ± 10.6	94.7 ± 12.4
SS-QOL	166.3 ± 21.8	188.2 ± 21.0	190.6 ± 21.0^*^	153.4 ± 31.6	161.8 ± 31.1	163.2 ± 29.8
BBS	40.8 ± 6.8	48.8 ± 6.4^*^	51.4 ± 3.3	36.7 ± 11.1	40.7 ± 10.8	43.1 ± 13.0
TBG	18.2 ± 4.1	23.1 ± 4.2	23.8 ± 3.8	18.0 ± 5.1	20.3 ± 5.0	21.7 ± 5.6
MAS UL	1.1 ± 0.7	0.8 ± 0.8	0.5 ± 0.7	0.8 ± 0.7	0.5 ± 0.7	0.4 ± 0.6
MAS LL	0.6 ± 0.7	0.2 ± 0.3	0.1 ± 0.3	0.4 ± 0.4	0.2 ± 0.4	0.2 ± 0.4

*SPAg, experimental group; CTRLg, control group; MBI, modified barthel index; SS-QOL, Stroke Specific Quality Of Life Scale; BBS, Berg balance scale; TBG, Tinetti balance and gait; MAS UL, modified Ashworth scale of the upper limb (determined as the average of the scores of elbow flexors, wrist flexors, and finger flexors of the affected upper limb); MAS LL, modified Ashworth scale of the lower limb (determined as the average of the scores of hip adductors, knee extensors, and ankle plantar flexors of the affected lower limb)*.

* *Significant for p < 0.05*.

Means ± standard deviations of clinical scales scores at T0, T1, and T2. The analysis of effectiveness revealed that, compared with baseline (T0), the improvement percentage in all clinical scale scores was greater in the SPAg group than the CTRLg (Table 3). Significant differences were found in the between-subject analysis of the BBS (*p* = 0.02) and SS-QOL (*p* = 0.03) scores.

**Table 3:** Percentages of effectiveness for in the two groups.

	**SPAg**		**CTRLg**	
	**Increase** **T1 vs. T0** **(%) ± SD**	**Increase** **T2 vs. T0** **(%) ± SD**	**Increase** **T1 vs. T0** **(%) ± SD**	**Increase** **T2 vs. T0** **(%) ± SD**
MBI	53.0 ± 30.9	69.0 ± 26.3	29.3 ± 13.6	58.0 ± 34.1
SS-QOL	27.4 ± 17.5^*^	29.7 ± 17.2	10.5 ± 7.5	17.9 ± 14.3
BBS	54.4 ± 31.1^*^	65.3 ± 26.1	26.8 ± 21.3	43.8 ± 31.6
TBG	55.0 ± 28.7	57.1 ± 28.3	35.5 ± 28.3	46.1 ± 32.6

*Mean ± standard deviation of percentage increase between T1 and T0 and between T2 and T0. The percentage increase was calculated as follows: [(T1 score – T0 score/maximal score – T0 score) × 100] and [(T2 score – T0 score/maximal score – T0 score) × 100]*.

*PSAg, experimental group; CTRLg, control group; MBI, modified Barthel index; SS-QOL, Stroke Specific Quality Of Life Scale; BBS, Berg balance scale; TBG, Tinetti balance and gait*.

* *Significant for p < 0.05 in the between-subject analysis*.

## Discussion

This is the first investigation of the effects of a sequential water-based preparatory approach compared with a conventional water-based therapy in stroke survivors. Our results confirm that both water-based approaches significantly improve motor functions in people who have suffered a stroke (21, 34, 35). These improvements can be attributed to the water environment, which partially supports the body, thus facilitating whole body movements (36).

From a clinical point of view, we found that the BBS score increased enough (i.e., from 40 to 48 points) to allow patients to pass from a level of assistance needed for walking to a level of safe independent walking only in the SPAg. Notably, this increase in the BBS score also indicates a significant reduction of the risk of falling, passing from a medium fall risk to a low fall risk (26, 37–39). Also for TBG, in the SPAg, the score increased (from 18 to 23), confirming a transition from a fall risk to a low fall risk (28). Interestingly, these improvements remained stable in both scale scores in the follow-up assessment. These results are relevant for several reasons. First, the SPA training improved dynamic motor function in a sample of patients with chronic stroke. This is important because walking functions tend to decline more at 6 months from stroke onset, after a transient initial improvement, and this deficit is associated with long-term disability and reduced QoL (40). Second, this improvement was achieved in a relatively short period of training (4 weeks, 2 sessions/week), showing that a challenging task-oriented and preparatory water-based therapy can be a useful complementary strategy for stroke survivors. Our results also show that the SPA is able to improve the QoL, and this improvement remained stable at follow-up. The significant improvement of the QoL could be related to the high percentage of SPAg improvement in balance and gait, which is reported in Table 3. The positive effect on balance functions could have facilitated participants' performance of the activities of daily living, facilitated participation in social activities, and favored indirect continued improvement at follow-up. However, these results should be taken with caution. In fact, the two patients who dropped out at follow-up in each group were among the most severe cases, and this could have partially inflated effectiveness at the follow-up assessment with respect to the T1 assessment. Finally, another potential bias concerns the use of effectiveness: the more the MBI score approaches the top, the more effectiveness increases. When we analyzed the rough percentages of improvement, we found an 11% increment in SPAg from T0 at T1 and a 17% increase from T0 to T2 with a further improvement of 6%, that is, about half of that obtained at T1. Quite similar results were obtained for CTRLg (6 vs. 4%) and for the BBS-score (for SPAg, 20 vs. 26%; and for CTRLg, 11 vs. 17%). Raw percentage and effectiveness are two faces of the same coin, and their values should lead to the same interpretation. We preferred to use effectiveness as the obtained percentage improvement of the maximum achievable improvement, in line with previous studies on stroke (41, 42), despite the caution needed in interpreting the data.

Surprisingly, spasticity significantly decreased in both groups. The mechanism by which hydrotherapy influences spasticity may be related to depressing the sensitivity of the muscle spindle and a decrease in skin sensitivity, thus reducing gamma fiber activity (43).

## Limitations

We acknowledge some limitations of the present study. First, our sample included different types of patients with stroke (i.e., hemorrhagic and ischemic, with damage in both right and left hemispheres). Another limitation is the absence of a multisensor instrumental assessment of dynamic balance and gait parameters (44–46). In addition to better tailoring the training for patients, the depth of the water should be adapted to their height. Another limitation is the lack of a third group treated with the land-based therapy. Future studies should take this into consideration in order to better evaluate the effectiveness of the SPA therapy.

## Conclusions

A sequential, preparatory water-based approach in the short term and midterm can be more effective than conventional aquatic therapy for improving chronic stroke survivors' motor functions and QoL.

## Data Availability Statement

The datasets generated for this study are available on request to the corresponding author.

## Ethics Statement

The studies involving human participants were reviewed and approved by Local Ethics Committee of Fondazione Santa Lucia IRCCS, (Protocol CE/PROG.728). The patients/participants provided their written informed consent to participate in this study. This clinical trial is registered on http://clinicaltrials.gov, under the following trial registration number: NCT04362202.

## Author Contributions

Conceptualization was carried out by MT, GT, AC, and MM. MI, MT, and DM implemented the methodology. SD, MI, and MT analyzed the data. SV, GT, and AC performed the investigation. MT, SD, and MI wrote and prepared the original draft. All authors reviewed and edited the manuscript.

## Conflict of Interest

The authors declare that the research was conducted in the absence of any commercial or financial relationships that could be construed as a potential conflict of interest. The reviewer AB declared a past co-authorship with one of the authors MI to the handling editor.
